# The Roles of Phospholipase A_2_ in Phagocytes

**DOI:** 10.3389/fcell.2021.673502

**Published:** 2021-06-10

**Authors:** Deepti Dabral, Geert van den Bogaart

**Affiliations:** Department of Molecular Immunology and Microbiology, Groningen Biomolecular Sciences and Biotechnology Institute, University of Groningen, Groningen, Netherlands

**Keywords:** phagocytosis, PLA_2_, pathogens, macrophages, trafficking

## Abstract

Phagocytic cells, such as macrophages, neutrophils, and dendritic cells, ingest particles larger than about 0.5 μM and thereby clear microbial pathogens and malignant cells from the body. These phagocytic cargoes are proteolytically degraded within the lumen of phagosomes, and peptides derived from them are presented on Major Histocompatibility Complexes (MHC) for the activation of T cells. Mammalian PLA_2_ isozymes belong to a large family of enzymes that cleave phospholipids at the second position of the glycerol backbone, releasing a free fatty acid and a lysolipid moiety. In human macrophages, at least 15 different PLA_2_ forms are expressed, and expression of many of these is dependent on pathogenic stimulation. Intriguing questions are why so many PLA_2_ forms are expressed in macrophages, and what are the functional consequences of their altered gene expression after encountering pathogenic stimuli. In this review, we discuss the evidence of the differential roles of different forms of PLA_2_ in phagocytic immune cells. These roles include: lipid signaling for immune cell activation, initial phagocytic particle uptake, microbial action for the killing and degradation of ingested microbes, and the repair of membranes induced by oxygen radicals. We also discuss the roles of PLA_2_ in the subsequent digestion of ingested phagocytic cargoes for antigen presentation to T cells.

## Introduction

A role for phospholipase A_2_ (PLA_2_) in phagocytic immune cells has been suggested since the 1980s (Waite et al., 1979; Scott et al., 1980). These phagocytic cells, such as macrophages, neutrophils, and dendritic cells, ingest particles larger than about 0.5 μM and thereby clear microbial pathogens and malignant cells from the body (Uribe-Querol and Rosales, 2020). Particularly macrophages and dendritic cells also present peptides derived from these phagocytosed antigens on major histocompatibility complex (MHC) types I and II to activate cytolytic and helper T cells, respectively, while lipids can be presented on CD1 (Burgdorf and Kurts, 2008). The process of phagocytosis and the subsequent processing of the ingested antigen are essential processes that contribute to both innate and adaptive immunity. As we will discuss in this review, evidence suggests that PLA_2_ is involved in all stages of pathogen encounter, from the initial signaling, to the uptake, degradation and presentation of the antigen.

The nomenclature of PLA_2_ is very complex and disordered as it follows a chronology reflecting their time-line of discovery (Dennis et al., 2011; Murakami et al., 2015; Ramanadham et al., 2015). Mammalian PLA_2_ isozymes belong to a large family, which have been assigned into groups I to XVI based on their primary amino acid sequence (Dennis et al., 2011; Murakami et al., 2015; Ramanadham et al., 2015). Based on their subcellular localization and Ca^2+^ requirement, these groups can be further categorized into six types called sPLA_2_, iPLA_2_, cPLA_2_, LPLA_2_, aiPLA_2_, and PAF-AH, and all these types are further categorized into subtypes (Table 1) (i) sPLA_2_ are Ca^2+^-dependent secretory PLA_2_ forms found in secretions, such as a tears, plasma and pancreatic juice (Dennis et al., 2011; Murakami et al., 2015; Ramanadham et al., 2015). These enzymes belong to groups I, II, III, V, IX, X, XI, XII, XIII, and XIV (Dennis et al., 2011; Murakami et al., 2015; Ramanadham et al., 2015). Within the sPLA_2_ subgroup, a capital English letter indicates the subtype of enzymes. For example, sPLA_2_-IIA is present in synovial fluid, while sPLA_2_-IID is present in pancreas and spleen (Dennis et al., 2011). (ii) cPLA_2_ are Ca^2+^-dependent cytosolic enzymes which belong to group IV, and its subtypes are cPLA_2_α, -β, -γ, -δ, -ε, and -ζ (Dennis et al., 2011; Murakami et al., 2015; Ramanadham et al., 2015). (iii) iPLA_2_ are Ca^2+^-independent cytosolic forms which belong to group VI, and its subtypes are iPLA_2_-β, -γ, -δ, -ε, -ζ, and -η (Dennis et al., 2011; Murakami et al., 2015; Ramanadham et al., 2015). Within the cPLA_2_ and iPLA_2_ types, the different subtypes are mostly indicated by Greek letters (Dennis et al., 2011; Murakami et al., 2015; Ramanadham et al., 2015). However, this nomenclature is not always consistently used and for example cPLA_2_-IVα and iPLA_2_-VIβ are also known as cPLA_2_-IVA and iPLA_2_-VIA. (iv) LPLA_2_ is a lysosomal PLA_2_ which belongs to group XV (Shayman et al., 2011; Shayman and Tesmer, 2019). (v) aiPLA_2_ are acidic Ca^2+^-independent PLA_2_ forms which belong to group XVI, and are better known as peroxisomal PLA_2_ (Sorokina et al., 2009; Fisher, 2018). (vi) platelet-activating factor hydrolases (PAFAH) belong to groups VIIA (also known as lipoprotein-associated PLA_2__;_ Lp-PLA_2_), VIIB and VIII (Stafforini, 2009; Dennis et al., 2011).

**TABLE 1:** Phospholipase A_2_ (PLA_2_) family and role of specific forms discussed in this review.

Type	Group	Sub types	Other name	Molecular weight (kDa)	Catalytic residue	References	PLA_2_ forms discussed
cPLA_2_	IV	α (A) β (B) γ (C) δ (D) ε (E) ζ (F)		60–114	Ser/asp	Dennis et al., 2011; Leslie, 2015	cPLA_2_-IVα in lipid signaling
iPLA_2_	VI	β (A) γ (B) δ (C) ε (D) ζ (E) η (F)	PNPLA9 PNPLA8 PNPLA6 PNPLA3 PNPLA2 PNPLA4	27–146	Ser/Asp	Dennis et al., 2011; Ramanadham et al., 2015	iPLA_2_-VIβ in supporting focal exocytosis at the phagocytic cup
sPLA_2_	I II III V IX X XI XII XIII XIV	A, B A, B, C, D, E, F A, B A, B		10–19	His/Asp	Dennis et al., 2011; Murakami et al., 2015; Murakami, 2017	sPLA_2_-IIA, V, X, XII as antimicrobial forms at the phagocytic cup and within closed phagosomes
LPLA_2_	XV			45	Ser/His/Asp	Dennis et al., 2011; Fisher, 2018	LPLA_2_ having bacteriocidal activity at the phagocytic cup and within phagolysosomes Also, its membrane repair mechanism
aiPLA_2_	XVI		Peroxiredoxin 6	25	Ser/His/Asp	Fisher, 2018	aiPLA_2_regulating NOX2 assembly, and its membrane repair mechanism
PAF-AH	VIIA VIIB VIII		Lp-PLA_2_, PLA_2_VII PAF-AH II PAF-AH I (PAFAH1B1 PAFAH1B2 PAFAH1B3)	26–45	Ser/His/Asp	Dennis et al., 2011; Karasawa and Inoue, 2015	Not discussed as relevant information was not found

**FIGURE 1:**
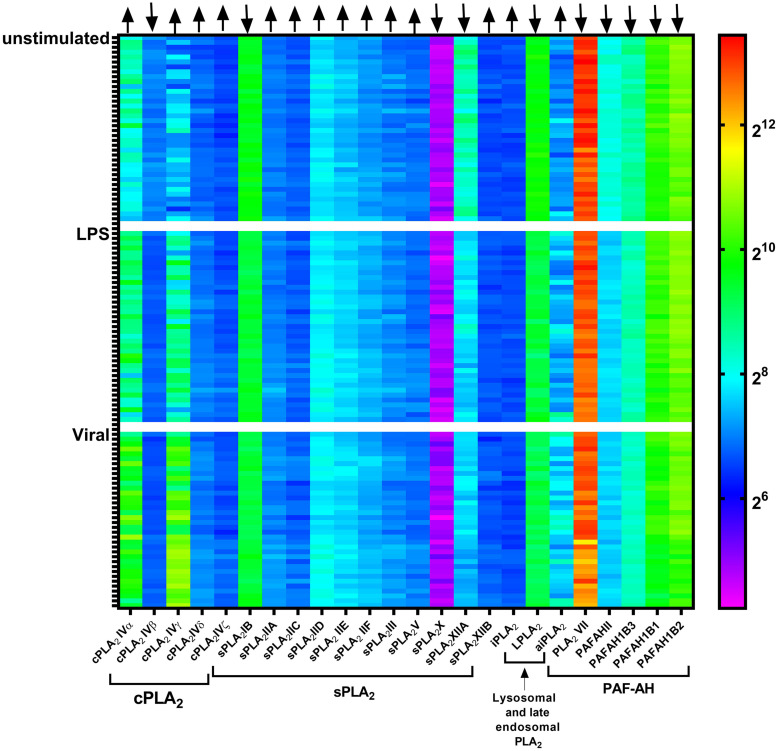
Gene microarray heatmap of phospholipase A_2_ (PLA_2_) gene expression in monocyte-derived dendritic cells from a published microarray study. Each column shows a PLA_2_ gene. Each row shows dendritic cells derived from an individual donor, either unstimulated or stimulated with LPS or the influenza virus (Viral). Microarray data is from the reference (Lee et al., 2014).

In human macrophages, 15 PLA_2_ forms are found at the transcript level, including sPLA_2_ (sPLA_2_-IID, -V, and -XIIA), cPLA_2_ (cPLA_2_-IV α, β, and γ), iPLA_2_ (iPLA_2_-VI β, γ, δ, ε, ζ, and η), LPLA_2_, LpPLA_2_, and aiPLA_2_ (Elstad et al., 1989; Rubio et al., 2015). Of these forms, LPLA_2_ and LpPLA_2_ carry a signal peptide required for cotranslational insertion into the ER and subsequent transport through the Golgi where they undergo *N*-glycosylation, while others are either cytosolic or membrane bound proteins (Dennis et al., 2011; Murakami et al., 2015; Ramanadham et al., 2015). Analysis of published gene expression data of blood monocyte-derived dendritic cells from 38 healthy individuals (Lee et al., 2014) revealed that the expression of some of these PLA_2_ forms is dependent on pathogenic stimulation (Figure 1). For example, the expression of cPLA_2_-IVα and -IVγ are upregulated, while sPLA_2_-XIIA and PAFAHII are downregulated upon stimulation of dendritic cells with lipopolysaccharide (LPS) or influenza virus. This information is important, because it shows that many forms of PLA_2_ undergo substantial up- or down-regulation upon pathogenic stimulation, suggesting that they have roles in the immune function of the phagocytes.

Indeed, although the roles of most PLA_2_ forms in macrophages and dendritic cells are largely unknown, literature shows that various PLA_2_ forms function during all the events that occur following the encounter of a pathogen: (i) the immune signaling by lipids carried out by cPLA_2_α, (ii) the focal exocytosis to support phagosome formation by iPLA_2_β, (iii) the killing of the microbe by sPLA_2_-V, and LPLA_2_ forms, and (iv) the repair of damaged phagosomal membranes inflicted by reactive oxygen species by LPLA_2_ and aiPLA_2_. In this review, we aim is to provide an overview of these functions of PLA_2_ forms.

## Lipid Signaling by cPLA_2_α

**FIGURE 2:**
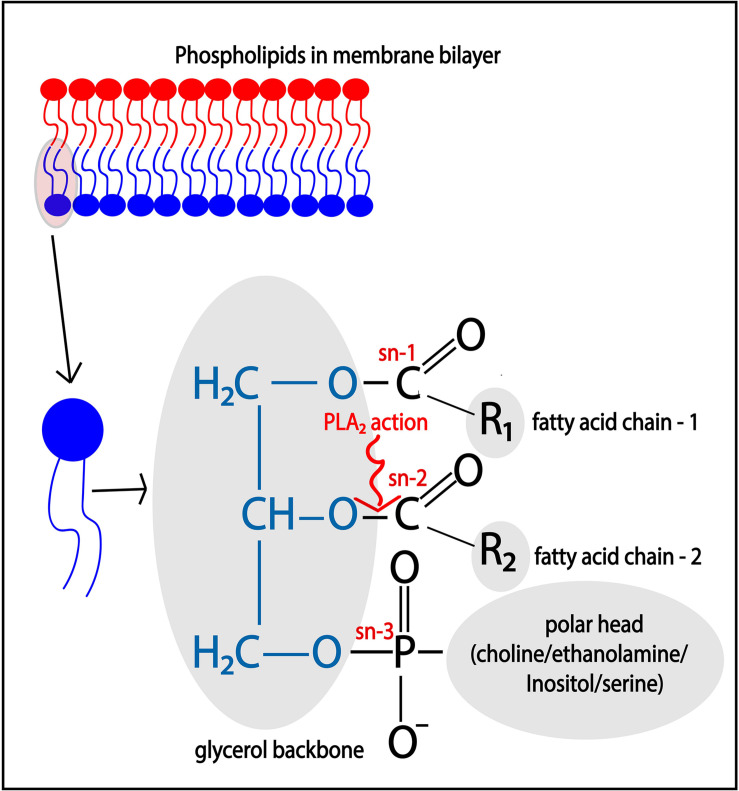
Catalytic action of PLA_2_. Membrane phospholipids such as phosphatidylcholine (PC)/phosphatidylethanolamine (PE)/phosphatidylinositol (PI), and phosphatidylserine (PS), are cleaved at the *sn*-2 position of the glycerol backbone by the action of PLA_2_, thereby releasing free fatty acid such as arachidonic acid.

Phospholipase A2 enzymes hydrolyze membrane phospholipids that are composed of a glycerol backbone esterified to two hydrophobic fatty acids tails at the *sn*- (stereospecifically numbered) 1 and 2 positions, and a hydrophilic head group at the *sn*-3 position (Figure 2). Phospholipids with the head groups choline, ethanolamine, serine, and inositol form the main classes of phospholipids and are called phosphatidylcholine (PC), phosphatidylethanolamine (PE), phosphatidylserine (PS), and phosphatidylinositol (PI), respectively. The catalytic action of PLA_2_ releases the free fatty acid from the *sn*-2 position, such as arachidonic acid (ARA; 20:4), docosahexaenoic acid (C22:6), oleic acid (C18:1), while lysophospholipids, such as lyso-phosphatidyl-choline/-ethanolamine/-inositol (LPC/LPE/LPI), remain esterified in the membrane.

Both the lyso-phospholipids and the free fatty acids produced by PLA_2_ can be bioactive molecules and/or form precursors for the generation of bioactive lipid hormones (Brash, 2001; Carneiro et al., 2013). PLA_2_ activity is especially important for the generation of a class of lipid hormones called eicosanoids. Eicosanoids are a broad family of oxygenated lipid compounds that include prostaglandins, thromboxanes, and leukotrienes. Eicosanoids are generated via non-enzymatic oxidation of ARA or by the action of enzymes, such as cyclooxygenase (COX), lipooxygenase (LOX), and cytochrome P450 (CYP) (Dennis and Norris, 2015; Gil-de-Gómez et al., 2020). Eicosanoids exert immunomodulatory functions and, depending on the species of eicosanoids, can have pro- or anti-inflammatory effects (Dennis and Norris, 2015). Upon encountering a pathogen, immune phagocytes produce elevated levels of eicosanoids and an increased amount of eicosanoids in circulation is considered a hallmark of inflammation (Dennis and Norris, 2015). Non-steroidal anti-inflammatory drugs (NSAIDs), such as ibuprofen, indomethacin, and asprin, target COX and PLA_2_ to alleviate pain, redness, and swelling associated with inflammation (Singh et al., 2005; Dennis and Norris, 2015).

Mammalian cells are rich in ARA, but this is mostly incorporated in phospholipids by esterification to the glycerol backbone at the *sn*-2 position. For example, human cultured platelets contain ∼30 μg esterified arachidonate per 10^9^ cells, which approximately corresponds to 5 mM, while free ARA is only present at ∼3 pmol per 10^6^ cells, corresponding to about 0.5–1 μM (Brash, 2001). However, the concentration of free ARA significantly increases upon pathogenic stimulation. For example, plasma concentrations in mice increase from ∼1 to ∼1.5 mM upon infection with *Salmonella pneumonia* (Eijkelkamp et al., 2018). Similarly, concentrations of ARA in the whole blood of sepsis patients increase significantly from ∼200 to ∼250 ng/ml (Bruegel et al., 2012). Phagocytosis also triggers ARA release *in vitro*, as shown for macrophages labeled with radioisotope labeled ARA (Waite et al., 1979; Gil-de-Gómez et al., 2020). This has been observed for a wide range of phagocytic cargoes, such as serum-opsonized zymosan, native zymosan, and live pathogenic bacteria (Adolph et al., 2012; Gil-de-Gómez et al., 2020). These studies indicate that pathogenic encounter results in an increased PLA_2_ activity resulting in the production of free ARA. The elevated ARA levels, in turn, promote the production of eicosanoids for inflammatory signaling. As will be discussed in more detail below, the elevated ARA levels might also facilitate the phagocytic process because supplementation of ARA to macrophage-like cell lines RAW264.7 and THP-1 accelerated phagocytosis, and potentiated their anti-microbial ability against intracellular microbes (Adolph et al., 2012; Eijkelkamp et al., 2018).

The most important PLA_2_ form involved in eicosanoid signaling is cPLA_2_α. cPLA_2_ isozymes are characterized by an N-terminal CalB domain for Ca^2+^ binding, an active site Ser-Asp dyad for lipid hydrolysis, and a C-terminal phospholipid-binding domain for interacting with membranes (Leslie, 2015). cPLA_2_ forms require mM concentrations of free Ca^2+^ for calcium-dependent membrane binding and activation (Dennis et al., 2011; Leslie, 2015). Members of this family include cPLA_2_α, -β, -γ, -δ, -ε, and -ζ, which vary in their molecular weight, tissue expression, and subcellular localization (Dennis et al., 2011; Leslie, 2015). These members have about 30% sequence homology and although they have overlapping activities, their functions are largely non-redundant. cPLA_2_α is the only PLA_2_ form that contains mitogen-activated protein kinase (MAPK) phosphorylation sites (S^505^ and S^727^) (Murakami, 2017). Thereby, infection-induced activation of the Ras and MAPK pathways results in the phosphorylation of cPLA_2_α (Zhou et al., 2003; Su et al., 2004). This phosphorylation results in increased activation of cPLA_2_α, which in turn leads to increased production of ARA (Dieter et al., 2002). Infection also induces cPLA_2_α transcription via transcriptional factors, such as nuclear factor κB (NF-κB), Krüppel-like factor, hypoxia-inducible factor (Hif), specificity protein 1 (Sp1), and c-Jun, that are well known to regulate immune cell activation (Su et al., 2004; Dennis and Norris, 2015; Lane et al., 2019). In line with this, transcriptomics analysis of human blood monocyte-derived dendritic cells revealed that both LPS and viral stimulation increase cPLA_2_α expression (Figure 1).

cPLA_2_α localizes in the cytosol in unstimulated RAW264.7 macrophage-like cells (Casas et al., 2006) and in mouse peritoneal primary macrophages (Gijón and Leslie, 1999). It translocates to a perinuclear membrane-rich area (likely the Golgi network) in response to chemically induced increases in PI (4,5)-bisphosphate [PI(4,5)P_2_] (Bechler et al., 2012) and Ca^2+^ concentrations at this location (Gijón and Leslie, 1999). A similar translocation has been observed in LPS-stimulated P388D1 macrophage-like cells (Balboa et al., 2003b; Shirai et al., 2005). In mouse peritoneal macrophages, cPLA_2_α and COX2 are both located close to the perinuclear Golgi network, which likely facilitates their functional coupling (Gijón and Leslie, 1999; Su et al., 2004). Thus, the increased expression and MAPK-mediated phosphorylation of cPLA_2_α upon encountering a pathogen, lead to its translocation to the Golgi, resulting in increased production of ARA (Gijón and Leslie, 1999). This mechanism underlies the increased production of eicosanoids upon infection.

However, in human monocyte-derived macrophages, immunofluorescence microscopy experiments revealed that GFP–cPLA_2_α also translocates to the phagocytic cup (Casas et al., 2010) and to zymosan containing phagosomes (Casas et al., 2009). This suggests that, next to lipid signaling, cPLA_2_α has additional roles in the phagocytic process. These additional roles seem to be independent of its catalytic activity, because mutated cPLA_2_α versions with an inactive catalytic domain showed no phagocytic defect in RAW264.7 macrophage-like cells, whereas mutation of the C2 domain, which is needed for membrane attachment, resulted in significantly less phagocytic ability (Zizza et al., 2012).

## iPLA_2_β Supports Focal Exocytosis for Pathogen Uptake

Unlike cPLA_2_ forms, group-VI iPLA_2_s do not require Ca^2+^ for their activity and membrane association (Dennis et al., 2011; Ramanadham et al., 2015). These are intracellular membrane-associated and cytoplasmic isozymes with a molecular weight ranging from 27 to 146 kDa (Dennis et al., 2011; Ramanadham et al., 2015). These isozymes are characterized by lipase (GXSXG) and nucleotide-binding (GXGXXG) consensus sequences (Dennis et al., 2011; Ramanadham et al., 2015). They also carry ankyrin repeats that mediate protein-protein interactions and this enables them to form homo-oligomers which is essential for their activity (Dennis et al., 2011; Ramanadham et al., 2015). Members of this family include iPLA_2_-β, -γ, -δ, -ε, -ζ, and -η. Notably, transcripts of iPLA_2_-β, -γ, -δ, -ε, -ζ, and -η have been detected in human monocyte derived macrophages (Rubio et al., 2015), iPLA_2_-β in human primary blood monocytes (Mishra et al., 2008) and the human monocyte cell line U937 (Tay and Melendez, 2004). Of these, only iPLA_2_-β (a cytoplasmic form), and -γ (membrane-associated form) isozymes are widely characterized (Dennis et al., 2011; Ramanadham et al., 2015).

iPLA_2_-β localizes to the ER–Golgi intermediate compartment (ERGIC), likely by associating to the cytosolic leaflet of the ERGIC membrane (Ben-Tekaya et al., 2010; Bechler et al., 2012). Studies in mammalian cell lines, such as HeLa, showed that iPLA_2_-β mediates the formation of membrane tubules that bridge between separate ERGIC clusters and thereby regulate intra-ERGIC trafficking (Ben-Tekaya et al., 2010; Bechler et al., 2012). However, the widely described role of iPLA_2_-β is a housekeeping function of remodeling phospholipids (Winstead et al., 2000; Ramanadham et al., 2015) that occurs in nearly all cellular membranes via two pathways: the Lands and the Kennedy (or *de novo*) pathways (Pasternak and Bergeron, 1970; Dabral and Coorssen, 2017). The Lands pathway maintains cellular homeostasis and operates in the presence of low concentrations of free ARA, which typically is the case in a resting cell, and iPLA_2_-β is a critical enzyme for this pathway (Kennedy and Weiss, 1956; Lands, 1958). In the Lands pathway, iPLA_2_-β generates free ARA, but most of it rapidly gets re-incorporated into phospholipids by first linking to Co-Enzyme A (CoA) by long-chain fatty acyl CoA synthetases. CoA-linked ARA then gets incorporated into PC by a CoA-dependent acyltransferase (Balsinde et al., 1997). Thus, iPLA_2_β-produced ARA is an intermediate of connected de-acylation and re-acylation reactions of membrane phospholipids. In unstimulated P388D1 macrophages, re-acylation dominates over de-acylation, which limits the presence of free ARA (Chilton et al., 1996; Balsinde et al., 1997), thereby also limiting eicosanoid production. The Kennedy pathway is a low-affinity mechanism that incorporates free ARA in membranes as triacylglycerol (TAG) and diacylglycerol (DAG) species (Kennedy and Weiss, 1956; Astudillo et al., 2012; Gil-de-Gómez et al., 2020). Thus, the incorporation of free ARA into membranes depends upon the concentration of free ARA: at low concentrations most ARA will be incorporated in phospholipids via the Lands pathway, while at higher concentrations it will be incorporated in TAG and DAG species via the Kennedy pathway. Since pathogenic stimulation results in increased activities of cPLA_2_ as described above, the rate of ARA hydrolysis exceeds that of its reincorporation into membrane phospholipids, leading to activation of the Kennedy pathway to esterify bulk ARA back in the membrane.

However, evidence suggests that iPLA_2_-β has a second role in supporting the phagocytic uptake. During phagocytosis, iPLA_2_-β translocates to the plasma membrane within 5 mins after particle engagement (Tay and Melendez, 2004). The catalytic activity of iPLA_2_-β is essential for particle uptake, as its inhibition using bromoenolactone (BEL)–a selective iPLA_2_-β and -γ inhibitor (Ramanadham et al., 2015)–leads to the polarized accumulation of electronlucent structures that appear as intracellular vesicles in the cytosol polarized toward the target attachment site in primary human monocytes (Lennartz et al., 1997). Moreover, enrichment of BEL-sensitive iPLA_2_ to F-actin rich pseudopods occurs in murine monocytes that are stimulated with monocyte chemoattractant protein-1 (MCP-1) (Mishra et al., 2008), a chemokine known to induce increased ARA release in human monocytes (Gschwandtner et al., 2019). Together, these findings indicate that vesicles carrying BEL-sensitive iPLA_2_ translocate to the plasma membrane at the site of the nascent phagosome, presumably to support membrane extension by promoting the local fusion of these vesicles with the plasma membrane (Bajno et al., 2000). These iPLA_2_ carrying vesicles might be of endosomal nature, as iPLA_2_ has been reported on endosomes (Karli et al., 1990; Mayorga et al., 1993, 1994). Alternatively or additionally, these might be secretory lysosomes, as BEL-sensitive iPLA_2_ is required for lysozyme secretion from these compartments (Balboa et al., 2003a).

## Anti-Microbial Roles of sPLA_2_

### Bactericidal Roles of sPLA_2_ Forms

Members of the sPLA_2_ group were first discovered in snake and bee venom, and in bovine pancreatic juice (Dennis et al., 2011). Humans express 10 catalytically active sPLA_2_ forms (IB, IIA, IIC, IID, IIE, IIF, III, V, X, and XIIA), and one inactive form (XIIB) (Murakami et al., 2015). Several of these forms are expressed in phagocytic immune cells. For instance, human primary macrophages express sPLA_2_-IIA, -IID, -V, -XIIA, and -XIIB (Anthonsen et al., 2000; Rubio et al., 2015), the macrophage-like cell line U937 expresses sPLA_2_-IID, -V, and -XIIA (Dennis et al., 2011), human monocyte-derived dendritic cells express sPLA_2_-IIA, -IIC, -IID, -IIE, -IIF, -III, -V, -X, -XIIA, and -XIIB (Anthonsen et al., 2000; Lee et al., 2014), and human monocytes express sPLA_2_-IIA, and -V (Anthonsen et al., 2000). sPLA_2_ forms are Ca^2+^-dependent and low molecular weight proteins (<10–19 kDa) which are secreted into the extracellular environment. They carry a His-Asp catalytic dyad, have a 6–8 intramolecular disulfide bonds, and contain a highly conserved Ca^2+^ binding loop (Dennis et al., 2011; Murakami et al., 2015). Intracellular sPLA_2_ is believed to be inactive and reside within the lumen of secretory vesicles and it becomes active after its secretion (Murakami et al., 2015).

Because its activity is largely confined to the extracellular environment, sPLA_2_ in principle can only cleave phospholipids that are exposed to the outside of the cell. Thus, sPLA_2_ enzymes can mainly hydrolyze lipids in the exoplasmic leaflet of the plasma membrane, and might hence act on the same cell that produces it in an autocrine manner, or paracrine on other cells. In addition, sPLA_2_ might act on other membranous extracellular structures such as extracellular vesicles, mitochondria, lipoproteins, and microbes (Dennis et al., 2011; Murakami et al., 2015). Both sPLA_2_-IIA and -V have specificity for phospholipids head-groups and cleave phosphatidylglycerol (PG) and phosphatidylethanolamine (PE) more efficiently than phosphatidylcholine (PC) (Dennis et al., 2011; Murakami et al., 2015). Because the outer monolayer of the mammalian plasma membrane is rich in PC, whereas bacterial membranes are mainly composed of PG and PE, sPLA_2_-IIA and -V are considered so-called bactericidal or inflammatory sPLA_2_s that primarily hydrolyze phospholipids of invading bacteria (Murakami et al., 2015).

Pathogenic stimulation promotes the secretion and expression of sPLA_2_-IIA and -V (Lee et al., 2014). In healthy human individuals, circulating sPLA_2_-IIA levels are low (∼3 ng/ml), and likely insufficient to kill bacteria (Grönroos et al., 2002; Movert et al., 2013). However, upon microbial infection, sPLA_2_-IIA production increases resulting in increased levels (250–500 ng/ml) in human and mouse serum, which are sufficient to kill microbe as shown by *in vitro* dose-response curves (Grönroos et al., 2002; Bruegel et al., 2012; Movert et al., 2013; Murakami et al., 2015). Unstimulated mouse peritoneal macrophages express sPLA_2_-V and an intracellular pool is present at the Golgi network and recycling endosomes (Balestrieri et al., 2006). Activation of macrophages results in the release of this sPLA_2_-V pool, as stimulating P388D1 macrophage-like cells with LPS for 6 h resulted in an increased localization of sPLA_2_-V in caveolin-rich vesicles near the perinuclear area (Balboa et al., 2003b). The pathogenic stimulation also increases the expression of sPLA_2_-V, as has been reported for LPS-stimulated primary human monocyte-derived macrophages (Rubio et al., 2015) and rat liver macrophages (Dieter et al., 2002). In line with this, transcriptomics analysis (Lee et al., 2014) also showed an upregulation in the expression of sPLA_2_-V in LPS and viral stimulated human monocyte-derived dendritic cells (Figure 1). However, sPLA_2_-XIIA seems down regulated upon pathogenic stimulation of monocyte-derived dendritic cells (Figure 1), but the functional significance of this is unknown.

The release of sPLA_2_-V might occur in a polarized fashion at the phagocytic cup, as it translocates from the Golgi and recycling endosomes to the forming phagosome in zymosan-stimulated mouse peritoneal macrophages (Balestrieri et al., 2006, 2009). This translocation of sPLA_2_-V from its juxtanuclear resting position to the forming phagosome was also observed within 5 mins after phagocytic cup formation in zymosan-stimulated peritoneal mouse macrophages (Balestrieri et al., 2006). Therefore, sPLA_2_-V at the phagocytic cup could potentially damage the attached microbe even before its internalization, especially since its activity will be promoted by the high extracellular calcium concentration (mM) (Mitsuishi et al., 2006; Balestrieri et al., 2009). Evidence supporting such a role in the killing of pathogenic microbes at the phagocytoc cup and/or later in closed phagosomes, comes from the finding that mouse peritoneal macrophages lacking sPLA_2_-V show delayed phagocytosis, delayed phagosomal maturation, and impaired *Candida albicans* killing (Lennartz et al., 1997). Similarly, human recombinant sPLA_2_-IIA, V, X, and XII substantially inhibited growth of *Staphylococcus aureus*, and *Listeria monocytogenes* in colony forming unit (CFU) assays (Koduri et al., 2002). However, the contribution of these sPLA_2_ forms in the killing of infectious pathogens *in vivo* is yet unknown.

### Bacterial Killing and Immune Modulation by LPLA_2_

Lysosomal PLA_2_ (LPLA_2_; group XV) is a glycoprotein and is highly mannosylated. The glycosylated form has a molecular weight of about 45 kDa (Shayman et al., 2011; Shayman and Tesmer, 2019). It carries a signal peptide for targeting to the ER, and is characterized by the conserved lipase motif AXSXG, containing an active site serine which is essential for its hydrolytic action (Shayman et al., 2011; Shayman and Tesmer, 2019). Its catalytic activity is calcium-independent, and it primarily localizes to the lumen of acidic lysosomes and late endosomes (Shayman et al., 2011; Shayman and Tesmer, 2019). Besides the canonical PLA_2_-mediated hydrolysis which releases a free *sn*-2 fatty acid from a phospholipid, LPLA_2_ is capable of transferring the fatty acid to the OH group at the C1 position of a short-chain ceramide in the so-called transacylase reaction. LPLA_2_ has an acidic pH optimum and is expressed in murine alveolar macrophages, RAW264.7 macrophage-like cells, and peritoneal mouse macrophages (Shayman et al., 2011; Shayman and Tesmer, 2019). The physiological roles of LPLA_2_ are still incompletely understood, and several functions have been proposed.

First, LPLA_2_ might regulate phospholipid metabolism, because phospholipids (i.e., PC, PE, and plasminogen-PE) were found to be accumulated in the alveolar lavage fluid of LPLA_2_ knock-out mice (Shayman and Tesmer, 2019).

Second, LPLA_2_ might mediate pathogen killing similar to sPLA_2_ described above, because LPLA_2_ is also released in the extracellular space of zymosan-stimulated mouse peritoneal and alveolar macrophages (Wightman et al., 1981; Abe et al., 2008). Also similar to sPLA_2_, LPLA_2_ translocates to forming phagosomes in RAW264.7 macrophage-like cells within 4 mins after particle engagement, and the fusion of lysosomes with the plasma membrane results in the exocytosis of LPLA_2_ (Sun et al., 2020). Because the V-ATPase at the plasma membrane pumps H^+^ ions across the membrane to the extracellular side, potentially lowering the local extracellular pH at the phagocytic cup, this might provide the required acidic microenvironment for secreted LPLA_2_ to attack the microbial cargo.

Third, LPLA_2_ might play a role in antigen presentation of lipids to T cells in CD1, because LPLA_2_ knock-out mice displayed lower T cell activation and recruitment to infected lungs (Shayman and Tesmer, 2019). Furthermore, *Mycobacterium* infected LPLA_2_ knock-out mice show an enhanced bacterial burden and a low recruitment of CD1-expressing cells to the infection site (Schneider et al., 2014). This role in lipid presentation is supported by the finding that LPLA_2_ can cleave bacterial cardiolipin, and the resulting lipid species are incorporated into the membranes of phagosomes and other organelles in *Mycobacterium bovis* infected murine macrophages (Fischer et al., 2001). Similarly, LPLA_2_ can cleave mycobacterial tetra-acylated glycolipid antigens (phosphatidyl-*myo-*inositol mannosides) into diacylated forms in THP-1 monocyte-like cells (Balboa et al., 2003a). Because cardiolipin carries four fatty acid chains, just as mycobacterial tetra-acylated glycolipids, LPLA_2_ appears to preferentially cleave tetra-acylated lipids (Fischer et al., 2001; Gilleron et al., 2016). Thus, current evidence suggests that LPLA_2_ processes microbial tetra-acylated lipids within phagolysosomes, and the resulting diacylated forms might be presented on CD1 molecules to stimulate T cells.

Fourth, LPLA_2_ could play a role in membrane repair, as explained in the next section.

## Membrane Repair by LPLA_2_ and aiPLA_2_

### Transacylase Activity of LPLA_2_ for Repair of Membrane Damage

In addition to its antimicrobial roles described in the previous section, LPLA_2_ also has a potential role in membrane repair. It can remove oxidized fatty acids at the *sn*-2 position of phospholipids and transfer these to ceramides in the transacylase reaction (Shayman and Tesmer, 2019). This ability of LPLA_2_ might be important, because in neutrophils, macrophages, and dendritic cells, the killing of ingested pathogens is a radical-mediated mechanism where the NADPH oxidase NOX2 generates large amounts of reactive oxygen species (ROS) in the lumen of phagolysosomes (Vulcano et al., 2004). These radicals not only damage the ingested pathogen, but also oxidize membranes of the host cell (Dingjan et al., 2016). Therefore, the host cell could need a mechanism to repair damage to the phagosomal membrane, particularly because polyunsaturated fatty acids, such as ARA, are highly susceptible to lipid peroxidation (Yin et al., 2011). LPLA_2_ might thus potentially limit or repair the damaging effects of ROS, by removing oxidized ARA from phospholipids and transfer it to ceramides. Supporting this transfer, ceramides containing long acyl chains such as ARA are high on mature phagosomes while ceramide synthase 2 expression and activity are high on early phagosomes in RAW264.7 macrophage-like cells (Pathak et al., 2018).

### Regulation of Oxidative Damage by aiPLA_2_

Acidic Ca^2+^-independent PLA_2_ (aiPLA_2_) has a molecular weight of ∼25 kDa and is better known as peroxiredoxin 6 (Fisher, 2011, 2018). It is expressed in mouse alveolar macrophages (Chatterjee et al., 2011) and mainly localizes to the cytosol where it has only low PLA_2_ activity due to its acidic optimum of pH 4.0 (Fisher, 2018). However, a fraction of aiPLA_2_ is also targeted to the lumen of lysosomes and late endosomes, in a manner depending on direct interactions with the chaperone 14-3-3ε (Sorokina et al., 2009; Fisher, 2018). Besides possessing PLA_2_ activity, aiPLA_2_ is a multifunctional enzyme that also possesses lyso-phosphatidyl acyltransferase and glutathione peroxidase activities (Fisher, 2011).

Because aiPLA_2_ binds to liposomes carrying oxidized lipids, and translocates to the plasma membrane in A549 cells following treatment with an oxidization agent (Manevich et al., 2009), membrane oxidation might promote association of aiPLA_2_ with the membrane. Hence, oxidized *sn-2* fatty acids might be replaced by the lyso-phosphatidyl acyltransferase activity of aiPLA_2_ (Fisher, 2018; Fisher et al., 2018). Additionally, aiPLA_2_ can reduce peroxidized phospholipids to their corresponding alcohol due to its glutathione peroxidase activity (Chatterjee et al., 2011; Fisher, 2018; Fisher et al., 2018). Therefore, similar to LPLA_2_, aiPLA_2_ can protect against oxidative damage due to its ability to (i) hydrolyze the *sn*-2 position peroxidized fatty acids to generate lyso-phospholipids, (ii) reacylate these lyso-phospholipids to form a new phospholipid, and (iii) convert oxidized phospholipids to their corresponding alcohol (Fisher et al., 2018). The physiological roles of aiPLA_2_ therefore include the repair of oxidized membranes (Chatterjee et al., 2011).

However, aiPLA_2_ also promotes ROS production by promoting the assembly of the NOX2 complex in alveolar macrophages (Chatterjee et al., 2011). As mentioned above, NOX2 generates ROS within the lumen of phagolysosomes, and also at the plasma membrane, in order to kill and degrade microbial pathogens (Burgdorf and Kurts, 2008; Uribe-Querol and Rosales, 2020). The assembly and activation of NOX2 occurs when MAPK phosphorylates cytosolic aiPLA_2_ at T^177^ which results in the translocation of aiPLA_2_ to the plasma membrane (Fisher et al., 2018). At the plasma membrane, aiPLA_2_ generates lyso-PC which in turn converts to lyso-phospatidic acid (LPA) by lysophospholipase D (Vázquez-Medina et al., 2016; Fisher, 2018). Binding of LPA to the LPA receptor-1 activates the small-GTPase Rac, which is a component required for the activation of the NOX2 complex (Fisher, 2018). In stark contrast, another component of the NOX2, p67^*phox*^, can bind to phosphorylated form of aiPLA_2_, and this inhibits its PLA_2_ activity (Fisher, 2018).

Thus, aiPLA_2_ has a dual function: it both repairs oxidation-induced membrane damage and promotes NOX2-mediated ROS formation. Perhaps at initial stages of pathogen recognition, the membrane damage by NOX2-produced ROS is still low. At this stage, unassembled p67^*phox*^ (i.e., not in the NOX2 complex) might negatively regulate aiPLA_2_ activity, as the need to repair damaged membranes is low. However, as more NOX2 is assembled and ROS production is increased, most p67^*phox*^ might be assembled in the NOX2 complex and no longer be available to inhibit aiPLA_2_. At this stage, aiPLA_2_ would be free to repair peroxidized phospholipids in the membrane.

aiPLA_2_ might also mediate the repair of membranes damaged by intracellular pathogens. The expression of aiPLA_2_ shows a biphasic response in *Brucella suis* infected RAW267.4 macrophages: aiPLA_2_ expression initially decreases until 10 h post-infection, after which it increases until 50 h post-infection (Wang et al., 2019). During the first phase, *B. suis* is non-replicative within phagosomes (Celli, 2019), whereas it becomes replicative in the second phase and this eventually leads to rupture of the phagosomal membrane (Celli, 2019; Wang et al., 2019). Therefore, the expression of aiPLA_2_ might initially be low because there is limited need for repair of membrane damage, whereas later its expression increases to perhaps repair the damaged phagosomal membrane. This mechanism is speculative and needs experimental support.

## Discussion and Concluding Remarks

**FIGURE 3:**
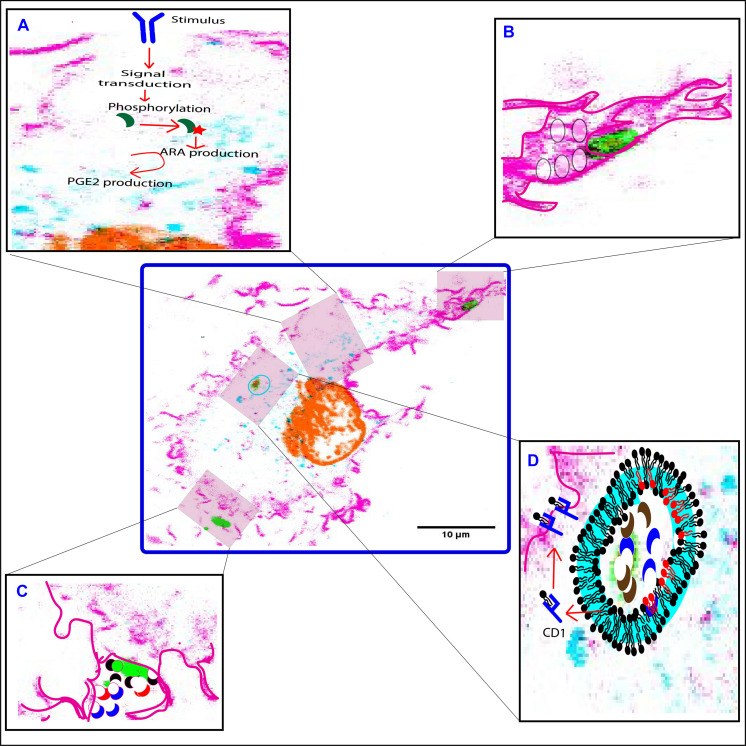
Functions of PLA_2_ forms at different stages of phagocytosis. **(A)** cPLA_2_α (green crescent) is phosphorylated in response to an extracellular pathogenic stimulus. This leads to translocation of cPLA_2_α to the perinuclear area, where it hydrolyzes membrane phospholipids to generate arachidonic acid (ARA) which in-turn gets metabolized to eicosanoids by the action of the COX-2 enzyme. **(B)** iPLA_2_ (not shown) translocates to the phagocytic cup, and facilitates fusion of secretory vesicles (translucent circles) to provide additional membrane required for the extension of the pseudopodia. **(C)** sPLA_2_-II/V (red and black crescent) and LPLA_2_ (blue crescent) are released at the phagocytic cup to degrade invading microbes (green). **(D)** LPLA_2_ (blue crescent) and aiPLA_2_ (brown crescent) work together to degrade and process microbes within the phagolysosome. The processed lipids are then loaded onto a CD1 molecule (blue structure) to be presented on the cell surface to activate T cells. Both LPLA_2_ and aiPLA_2_ repair peroxidized lipids of the phagosomal membrane that might occur due to increased NOX2 activity. The repaired phospholipids are shown in red. F-actin is pink, DNA is orange, LAMP1 is turquoise and LPS is green. Highlighted pink boundary in insets **(B–D)** shows pseudopodia, phagocytic cup, and plasma membrane, respectively. Highlighted turquoise boundary at the phagosome membrane is to show recruited LAMP1.

As discussed above, PLA_2_ forms have different effects on phagocytosis: aiPLA_2_, LPLA_2_, and sPLA_2_ forms mainly act on the luminal and/or extracellular leaflet of the (nascent) phagosomal membrane and play roles in the killing of the pathogen and the repair of the membrane from oxidative damage. In contrast, iPLA_2_ and cPLA_2_ forms mainly act on the cytosolic leaflets of the plasma membrane, phagosomes and other organelles, and play roles in eicosanoid signaling and regulation of organellar trafficking. These roles are summarized in Figure 3. In addition, since the products from PLA_2_ hydrolysis directly alter the physicochemical properties of the membrane, PLA_2_s might also directly affect the phagocytic process. ARA and LPC have negative and positive spontaneous curvatures, respectively, and thereby can directly stabilize or destabilize membrane assemblies (Chernomordik et al., 1995). Increased levels of ARA in the *cis* leaflets of merging bilayers (i.e., the cytoplasmic leaflet of the plasma membrane and the outer leaflet of the organellar membrane) promote fusion, while more LPC blocks fusion (Chernomordik et al., 1995). In contrast, more ARA in the *trans* leaflets (i.e., outer leaflet of the plasma membrane and luminal leaflet of the organellar membrane) of merging bilayers blocks fusion, while more LPC promotes fusion (Chernomordik et al., 1995). Therefore, membrane fusion might be both promoted and inhibited by these PLA_2_ metabolites depending on the site of PLA_2_ action, the PLA_2_ substrates, and downstream metabolism of the PLA_2_ products. Moreover, the fusion of intracellular membrane compartments at the phagocytic cup results in the delivery of more PLA_2_s to the nascent phagosome, including cPLA_2_, sPLA_2_, LPLA_2_, aiPLA_2_, and iPLA_2_ forms. This local delivery of more PLA_2_ at the nascent phagosome might thus modulate the phagocytic process by either facilitating or inhibiting the membrane reshaping required for the membrane wrapping and internalization of the phagocytic cargo.

In addition to these spontaneous effects on membrane fusion by the physicochemical properties of PLA_2_ products, PLA_2_ might potentially also affect phagocytosis by affecting soluble *N*-ethylmaleimide-sensitive factor attachment protein receptors (SNARE)-mediated membrane fusion. In animal cells, all organellar membrane fusion (except mitochondrial fusion) is mediated by members of the SNARE protein family (Hong, 2005). Cognate SNARE proteins in both the vesicular and target membranes, called v- and t-SNAREs, engage and “zipper” from their N-terminal toward their C-terminal termini, thereby forming a tight alpha-helical coiled-coil bundle that overcomes the energy barrier of membrane fusion (Dingjan et al., 2018). In mouse chromaffin cells, the transmembrane domain of the v-SNAREs VAMP2 is affected by the PLA_2_ metabolites LPC and oleic acid in a leaflet specific manner that either promotes or inhibits fusion pore formation and expansion, hence affecting neurotransmitter release (Dhara et al., 2020). In line with this, the administration of LPC and oleic acid to the cell culture medium, which increases their concentrations in the outer leaflet of the plasma membrane, accelerated and deaccelerated nurotransmitter release, respectively (Dhara et al., 2020). In contrast, the intracellular administration of LPC and oleic acid by microinjection, which increased their concentration in the inner leaflet of the plasma membrane, blocked and promoted membrane fusion, respectively (Dhara et al., 2020). Similar results were also obtained in homotypic fusion of cortical vesicles isolated from eggs of the sea urchin, in which increasing the LPC concentrations in the outer leaflets of these vesicles by external LPC blocked fusion (Dabral and Coorssen, 2019). However, the PLA_2_ products might not only affect the membrane fusion, but also upstream events as the external application of ARA also affected the SNARE dependent docking or priming in homotypic fusion of cortical vesicles (Dabral and Coorssen, 2019). Because many SNARE proteins are both functionally and structurally homologous, and since many SNARE proteins are involved in endosomal trafficking and phagocytosis (Dingjan et al., 2018), similar interactions with PLA_2_ metabolites could potentially inhibit or promote membrane fusion in phagocytosis as well.

Whereas the roles of the different PLA_2_ types in phagocytes are fairly well established, as discussed in this review, a main open question is why immune cells express so many different members of each PLA_2_ group. For example, human monocyte derived macrophages express three forms of sPLA_2_ (sPLA_2_-IID, -V, and -XIIA), three forms of cPLA_2_ (cPLA_2_IV-α, -β, and -γ) and six forms of iPLA_2_ (iPLA_2_VI-β, -γ, -δ, -ε, -ζ, and -η) (Rubio et al., 2015). While these different types have non-overlapping functions in eicosanoid signaling, membrane repair, organellar trafficking and pathogen killing, it is largely unknown why multiple members of each different type are expressed. PLA_2_ members from each type perhaps are essential for carrying out the same critical processes. Hence, multiple genes coding for these essential enzymes might ensure the functional redundency in the absence of one of the forms, thereby increasing the fidelity of the immune response. It might also be that these different members have different specificities for headgroups and acyl chains, and/or they might have different subcellular localizations. Thereby, the various PLA_2_ subgroup members might have non-overlapping roles, and could for instance act during different stages of the phagocytic process: antigen recognition, formation of the phagocytic cup, particle internalization, and maturation of the phagosome into a phagolysosome. Supporting such non-overlapping roles, is the finding that expression of different subtype members of cPLA_2_ is differently regulated upon pathogenic stimulation: transcript levels of cPLA_2_-IVα and -IVγ are upregulated, whereas cPLA_2_-IVβ is downregulated (Figure 1) (Lee et al., 2014).

Another open question is whether microbial PLA_2_ forms also modulate phagocytic process. Several bacterial pathogens express PLA_2_ enzymes as virulence factors. For example, PLA_2_ activity of *Helicobacter pylori* is responsible for the degradation of the gut mucosal barrier (Baj et al., 2020). The putative PLA_2_ protein RV3091 of *Mycobacterium tuberculosis* is involved in the phagosomal escape of this pathogen (Cui et al., 2020) and a secreted form of PLA_2_ by *Toxoplama gondii* contributes to its replicative cycle (Cassaing et al., 2000). These bacterial PLA_2_s are structurally similar to mammalian sPLA_2_ forms and thus are evolutionary conserved (Murakami et al., 2015). Hence, we presume that within the lumen of a phagosome or at the nascent phagocytic cup, both host and microbial PLA_2_ likely engage in a contest to cleave the lipids of the bacterial and host membranes, respectively, but the functional impact of such engagement in phagocytic cells is yet unexplored.

Experimentally, it is difficult to discriminate the actions of the different PLA_2_ forms, both from host and microbial origin, because many PLA_2_ inhibitors are not entirely specific for one specific species of PLA_2_. Moreover, all PLA_2_ forms act on membrane phospholipids, and lipidomics approaches therefore do not readily allow the assignment of the results to a single species of PLA_2_. The potential functional redundancy of PLA_2_s also make them difficult to study, as knockout or knockdown might not always result in a clear phenotype. Nevertheless, we expect that the side-by-side comparison of mammalian cell lines with specific knockouts of one or more PLA_2_ forms, for instance using CRISPR technology, coupled with MS based organellar lipidomics and high resolution microscopy, will enable to address this functional redundancy and better delineate the unique and overlapping roles of the different PLA_2_ forms.

As summarized in Figure 3, PLA_2_ is important for the clearance of pathogens, because it (i) triggers eicosanoid signaling shaping the downstream immune response, (ii) stimulates or inhibits the phagocytic process, (iii) directly supports the killing of pathogenic microbes, and (iv) repairs oxidation induced phagosomal membrane damage. Therefore, the understanding of the expression, membrane association, substrate specificity and the associated immunomodulatory actions of PLA_2_ forms in regulating the immune response in phagocytic cells is critical, as this might also lead to new therapeutic approaches to combat microbial infections.

## Author Contributions

DD conceptualize and drafted the original draft. GB reviewed and provided critical feedback. Both authors equally contributed to the final preparation of the manuscript.

## Conflict of Interest

The authors declare that the research was conducted in the absence of any commercial or financial relationships that could be construed as a potential conflict of interest.
